# End-of-life medical decisions in France: a death certificate follow-up survey 5 years after the 2005 act of parliament on patients’ rights and end of life

**DOI:** 10.1186/1472-684X-11-25

**Published:** 2012-12-03

**Authors:** Sophie Pennec, Alain Monnier, Silvia Pontone, Régis Aubry

**Affiliations:** 1 Institut National d’Etudes Démographiques, 133, boulevard Davout, 75980, Paris cedex 20, France; 2 Australian Demographic and Social Research Institute, Australian National University, Canberra, Australia; 3 Robert Debré Hospital, Assistance Publique-Hôpitaux de Paris, Paris, France; 4 Observatoire National de la Fin de Vie, Paris, France; 5 Jean Minjoz Hospital, Besançon, France

## Abstract

**Background:**

The “Patients’ Rights and End of Life Care” Act came into force in France in 2005. It allows withholding/withdrawal of life-support treatment, and intensified use of medications that may hasten death through a double effect, as long as hastening death is not the purpose of the decision. It also specifies the requirements of the decision-making process. This study assesses the situation by examining the frequency of end-of-life decisions by patients’ and physicians’ characteristics, and describes the decision-making processes.

**Methods:**

We conducted a nationwide retrospective study of a random sample of adult patients who died in December 2009. Questionnaires were mailed to the physicians who certified/attended these deaths. Cases were weighted to adjust for response rate bias. Bivariate analyses and logistic regressions were performed for each decision.

**Results:**

Of all deaths, 16.9% were sudden deaths with no information about end of life, 12.2% followed a decision to do everything possible to prolong life, and 47.7% followed at least one medical decision that may certainly or probably hasten death: withholding (14.6%) or withdrawal (4.2%) of treatments, intensified use of opioids and/or benzodiazepines (28.1%), use of medications to deliberately hasten death (i.e. not legally authorized) (0.8%), at the patient’s request (0.2%) or not (0.6%). All other variables held constant, cause of death, patient's age, doctor’s age and specialty, and place of death, influenced the frequencies of decisions. When a decision was made, 20% of the persons concerned were considered to be competent. The decision was discussed with the patient if competent in 40% (everything done) to 86% (intensification of alleviation of symptoms) of cases. Legal requirements regarding decision-making for incompetent patients were frequently not complied with.

**Conclusions:**

This study shows that end-of-life medical decisions are common in France. Most are in compliance with the 2005 law (similar to some other European countries). Nonetheless, the study revealed cases where not all legal obligations were met or where the decision was totally illegal. There is still a lot to be done through medical education and population awareness-raising to ensure that the decision-making process is compatible with current legislation, the physician's duty of care and the patient’s rights.

## Background

The annual number of deaths in France, ranging between 500,000 and 600,000, has varied relatively little since World War II, with the decline in mortality rates being offset by population growth. However, as the large post-war cohorts grow older, the numbers of deaths will increase considerably (to 770,000 by 2050). Whereas just after World War II most people died at home, in France, as in other developed countries, most now die in hospital.

These changes are bound to affect the way death is seen and experienced, and to influence future health policy and medical practices. As medical progress increasingly prolongs the life expectancy of the chronically ill, end-of-life questions such as place of death, palliative care, decisions to withdraw or reduce active treatment, staff resources, the role of the family in end-of-life care and demand for euthanasia are becoming ever more critical.

End-of-life legal provisions vary widely across Europe. In France, the Act of 22 April 2005 on Patients’ Rights and End-of-Life Care [1] introduced three main measures: Firstly, it prohibits “unreasonable obstinacy” and therefore the continuation of futile medical treatments. Secondly, it strengthens the right of access to palliative care for any person whose condition requires such care, and recognizes that, under certain conditions, pain and symptom relief may require drugs that, at high doses, may have the unintended effect of shortening the patient’s life. Thirdly, it strengthens the principle of patient autonomy and of discussion with the patient. If the patient is not competent, the end-of-life medical decision must be taken after discussion with a trusted third party or surrogate (if the patient has named one), and the family, if any, and after consultation with medical staff or colleagues.

Nevertheless, legalising euthanasia remains a highly controversial topic in the public and political arena, as seen during the 2012 presidential election.

In Europe, various surveys [2-5] have shown that in order to better understand end-of-life conditions, it is important to study the medical decisions taken prior to death. In France, the only surveys on end-of-life decisions conducted until now focused on deaths in hospital or emergency wards [6-9]. The survey *Fin de Vie en France* (“End of life in France”), conducted in 2010, concerned all deaths, regardless of cause or place (hospital, home, nursing home…). It provides an overview of end-of-life care in France that can be used as a baseline for assessing future developments.

This paper focuses on the medical decisions relating to end-of-life care in France. It looks at how the decisions varied according to the person's and physician's characteristics. It also investigates the extent to which these decisions comply with the 2005 law.

## Methods

### Retrospective survey of physicians

As in previous European surveys [10], we conducted a retrospective survey on a sample of deaths where the respondents were the certifying physicians.

This sample of 14,999 deaths was selected by Inserm-CepiDc (Centre d’épidémiologie sur les causes médicales de décès) using a systematic random procedure. We ensured that it was representative (in terms of age, sex, place of death and region of death) of the 47,872 persons aged 18 and over who died in France in December 2009. Stratification by cause of death (a proxy for the likelihood of an end-of-life decision) was not possible because of the delay in registration of causes of death.

For each death, we identified the certifying physicians on the death certificates and we mailed them the questionnaire with instructions for replying. Physicians could respond either by post (with paid-reply envelope) or online. These two response channels involved trusted third parties (virtual for Internet responses) to ensure medical confidentiality with regard to the deceased and the anonymity of both the deceased persons and the physicians taking part in the study [11]. Three follow-up letters were sent and a follow-up phone call was made.

We also conducted a telephone survey on a sample of 620 non-responding physicians to ensure that the results were representative. We recorded their socio-demographic profiles and their reasons for non-response.

### Questions and variables

The questionnaire was based on the Eureld survey questionnaire [10] but was adapted to take account of the French legal context and of the results of preliminary tests. It comprised 113 questions (see Additional file 1). End-of-life medical decisions and the decision-making process were explored in the middle part of the questionnaire after questions about the end-of-life context (characteristics of the deceased person, physician, place of death, whether palliative care had been provided). Another section comprised questions on the physician's feelings about the death. The last section asked the physicians whether they habitually respond to surveys and what made them decide to respond to this particular survey if such was the case.

The key questions about end-of-life medical decisions were (see Additional file 2) (1) whether first of all everything was done to prolong the patient’s life (2) whether a treatment of any kind was withheld; (3) whether a treatment of any kind was withdrawn; (4) whether a treatment to alleviate the symptoms was intensified (opioids, benzodiazepines and/or any other treatment) and (5) whether a medication was administered to the patient to deliberately end his/her life. For questions (2) to (4), three sub-questions investigated the physician's intention: (a) did he/she know that his/her decisions could hasten the death (b) did he/she take the decision with the explicit intention of hastening the death and (c) did he/she consider the decision to have hastened the death. We classified the answers to these questions to ensure maximum similarity with the EURELD classification of medical end-of-life decisions (as published in Van den Heide [4]): when one of questions (2) (3) and one of their sub-questions (a) (b) were answered *yes*, the case was classified as “*non treatment decision*”; when question (4) and one of its sub-questions (a) (b) were answered *yes*, the case was classified as “*intensification of alleviation of symptoms with possible life shortening effect*”; when question (5) was answered *yes*, we classified the case as “*using a medication to deliberately hasten death*”, *differentiating between treatment at the patient*’*s explicit request*, *administration by the patient him*/*herself in* “*physicianassisted suicide*” *or administration by a nurse or a physician*. When questions (2) to (5) were answered *no* and question (1) was answered *yes*, the case was classified as “*lifeprolonging decision*”; when questions (1) and (5) were answered *no* and the sub-questions (a) or (b) to questions (2) (3) (4) were answered *no*, we classified the case as “*no decision*”. When questions (2), (3) or (4) were answered “*yes*” and sub-questions (a) and (b) were answered “*no*” or “missing”, the case was classified as “*medical decision without any intention regarding death*”.

When more than one question (1) to (5) had been answered *yes*, the decision with the most explicit intention took precedence over other decisions. In cases of similar intention, question (5) took precedence over question (4), which took precedence over question (3), which took precedence over question (2).

We define as sudden deaths those deaths that the physician considered as sudden and unexpected and for which he/she cannot give any information about the end of life. Some deaths were qualified as sudden by the physician, although he/she was able to give information about end-of-life decisions, and such a decision did exist in 60% of those cases. We therefore included them in our study of “non sudden deaths”.

Information on the person's ability to express his/her wishes was based on what the physician had noted from his/her discussion of the decision with the person, and on the reasons put forward for not having had such a discussion. Some persons were judged not competent to be involved in a discussion about their wishes, and some further persons, after taking part in such a discussion, were judged not competent to appreciate their situation and/or to decide for themselves. We did not have any clear information about the persons’ abilities in other cases. We considered that the remaining persons were competent.

### Participation

Among the entire initial sample of 14,999 deaths, 646 of the certifying physicians could not be identified. No physician was asked about more than four deaths, even if they had certified more. In all, 14,080 questionnaires were sent to 11,828 certifying physicians (of whom 14% had signed more than one death certificate). 461 questionnaires did not reach the addressee owing to postal problems or typing errors; 608 responses did not include a completed questionnaire (e.g., physician no longer practising or not the attending physician). 5,217 completed questionnaires were received, giving an overall participation rate of 40% [12]. Only a quarter of responses were obtained online even though respondents were given a full guarantee of anonymity with both collection modes. The results presented are based on 4,891 questionnaires since 327 concerned deaths outside the observation period.

### Quality control and weighting

The characteristics of the 4,891 deaths in the survey are very close to those of all deaths in December 2009. There is a slightly higher percentage of deaths in public hospitals (55% versus 50%) and a correspondingly lower percentage at home, in private hospitals and retirement homes. Cancers and infectious diseases are slightly over-represented, and cardio-vascular diseases and deaths from external causes are under-represented.

The answers were weighted for non-response bias according to the number and distribution of the reference variables used to select the initial sample of death certificates: age and sex of the deceased, place of death and region of residence.

### Statistical analysis

The categorical data were described using frequencies and percentages. Univariate and bivariate analyses were tested with the exact Fisher test instead of a standard Chi square because of the low numbers in some categories. It tests the relation between a variable and a particular medical decision, i.e. whether the observed distribution of a variable for a particular medical decision is different from cases without this medical decision. Logistic regressions were performed for each medical decision with more than 150 observed cases, taking into account both patients' and physicians' characteristics. All tests were performed at a significance level of 1%. Logistic regressions (not shown) were performed to determine the variables or characteristics that remain significant, all other variables held constant. The results section focuses on the significant effects of these variables. The statistical analyses were performed using the SAS Version 9.2 statistical software package.

### Ethics

This survey was approved by the *Comité Consultatif sur le Traitement de l*’*Information en Matière de Recherche dans le Domaine de la Santé* (CCTIRS) in January 2010 and authorized by the *Commission Nationale de l*’*Informatique et des Libertés* data protection committee (CNIL, - authorization No. 1410166 at sitting 2010–107 of 15 April 2010).

## Results

### End-of-life medical decisions

We had to exclude 168 cases owing to missing data. Sudden deaths (n=798) amounted to 16.9% of the total (Table 1). For 2,252 non-sudden deaths, one or more decisions were made that possibly or certainly hastened death. For almost half of these deaths, there were two or more decisions. In 34% of all deaths, a life-prolonging treatment was withheld; in 11% it was withdrawn. In 29% of cases alleviation of pain and/symptoms was intensified and in 0.8% a medication was administered deliberately to hasten death.

**Table 1:** Frequency of all the different end-of-life medical decisions

	**All decisions**				**Most important decision**		
	**Weighted data**				**Weighted data**		
	**N**	**Percentage**	**95% Confidence interval**	**Unweighted data N**	**N**	**Percentage**	**95% Confidence interval**
							
**Sudden death**	798	16.9	15.8-18.0	789	798	16.9	15.8-18.0
**Medical decision without any intention regarding death**							
*Life*-*prolonging treatment*	1513	32	30.7-33.4	588	576	12.2	11.3.13.2
*Treatment withheld*	325	6.9	6.2-7.6	140	140	3	2.5-3.5
*Treatment withdrawn*	86	1.8	1.4-2.2	15	15	0.3	0.2-0.5
* Intensification of treatment to alleviate symptoms with opioids or benzodiazepines*	386	8.2	7.4-8.9	252	246	5.2	4.6-5.8
*Intention of treatment to alleviate symptoms with medications other than opioids or benzodiazepines*	199	4.2	3.6-4.8	97	97	2.1	1.7-2.5
*None of the above*	599	12.7	11.7-13.7	571	599	12.7	11.7-13.7
**Medical end**-**of**-**life practice that possibly or certainly hastened death**				2239	2252	47.7	
Treatment withheld	1594	33.7		691	688	14.6	13.6-15.6
*Knowing that the decision may hasten the death*	1526	32.3	31.0-33.6	657	655	13.9	12.9-14.9
*With the intention of hastening death*	68	1.4	1.1-1.8	34	33	0.7	0.5-0.9
Treatment withdrawn	531	11.2		202	199	4.2	3.6-4.8
*Knowing that the decision may hasten the death*	465	9.8	9.0-10.7	162	161	3.4	2.9-3.9
*With the intention of hastening death*	66	1.4	1.1-1.7	40	38	0.8	0.6-1.1
Intensification of treatment to alleviate pain and/or symptoms (opioids/benzodiazepines)	1381	29.2		1346	1327	28.1	26.8-29.4
*Knowing that the decision may hasten the death*	1324	29.0	26.7-29.3	1306	1288	27.3	26.0-28.5
*With the intention of hastening death*	57	1.2	0.9-1.5	40	39	0.8	0.6-1.1
Use of a drug to deliberately end life*	38	0.8	0.5-1.1	38	38	0.8	0.5-1.1
*At patient*’*s request*	11	0.2	0.1-0.4	10	11	0.2	0.1-0.4

Sudden deaths: deaths declared “sudden and unexpected” and on which the physician has no information about the end of life.

The weighted percentages are weighted for non-response bias.

All decisions = every affirmative answer to questions about medical decisions.

Most important decision = when more than one decision is observed, the one with the most explicit intention (or awareness) of hastening death (see classification details in Methods).

* no doctor assisted suicide have been observed.

Missing values: 168.

Considering only the most important decision for each death, the proportion of cases with administration of medication to deliberately hasten death does not change (0.8% of all deaths). Of these 38 decisions, 11 were at the patient’s request.

The physician reported increasing opioid and/or benzodiazepine doses in another 28% of all deaths. Withdrawal of life-prolonging treatment was decided in 4% of all deaths, and life-prolonging treatment was withheld in another 15% of all cases. These medical decisions were made with the explicit intention to hasten death in 0.8%, 0.8%, 0.7% of cases, respectively.

In all, considering only the most important medical decisions, 3.1% of all deaths followed a decision to hasten death.

For 12.2% of all deaths, the sole reported medical decision was doing everything possible to prolong life, and for 12.7% no decision was made (no decision to do everything to prolong life and none of the other decisions mentioned in the questionnaire). In 11% of cases a medical decision without any intention regarding death was reported.

### End-of-life medical decisions by patient's and physician's characteristics

The pathology (cause of death and incurability) and age of the deceased were decisive in end-of-life decisions. The frequency of end-of-life decisions varies from 71% for cancer to 41% for cardio-vascular diseases. For cancer patients, 3 in 4 end-of-life medical decisions consisted of intensifying alleviation of symptoms; for respiratory diseases, end of life decisions were more diverse, with intensification of alleviation of symptoms in 40% of cases and withholding a treatment in 50%. It is difficult to interpret the variations in administration of a drug to deliberately end life owing to the small numbers concerned (Table 2).

**Table 2:** Frequency of all the different medical end-of-life decisions in France by patients’ characteristics (non-sudden deaths)

	**Life prolonging treatment**		**Withholding treatment**		**Withdrawing treatment**		**Intensification of alleviation of symptoms**		**Administration of a medication to deliberately hasten death**		**No decision**		**Total deaths**
	**n**	**%**	**n**	**%**	**n**	**%**	**n**	**%**	**n**	**%**	**n**	**%**	**n**
**Gender**													
Man	302	15.62	335	17.32	105	5.43	641	33.15	20	1.02	531	27.46	1934
Woman	274	13.74	354	17.78	94	4.72	686	34.45	18	0.89	566	28.42	1991
*p value*		*0*.*0865*		*0*.*7323*		*0*.*3214*		*0*.*4289*		*0*.*6790*		*0*.*5478*	
**Age**													
18-49	37	24.82	14	9.41	7	4.71	50	33.62	1	0.56	40	26.89	149
50-69	139	19.79	74	10.52	43	6.11	268	38.08	11	1.63	168	23.87	704
70-79	113	14.63	128	16.63	42	5.46	294	38.20	7	0.90	186	24.17	770
80-89	204	13.05	309	19.79	79	5.06	486	31.13	16	1.04	467	29.92	1561
90+	83	11.30	163	22.07	29	3.93	227	30.74	2	0.27	234	31.69	738
*p value*		<*0*.*0001*		<*0*.*0001*		*0*.*4066*		*0*.*0003*		*0*.*1118*		*0*.*0008*	
**Place of death**													
At home	104	15.60	100	14.96	22	3.29	150	22.44	9	1.37	283	42.34	668
Public hospital	334	15.67	393	18.44	121	5.68	793	37.21	16	0.76	474	22.24	2131
Private hospital	59	14.97	56	14.15	23	5.81	156	39.41	8	1.92	94	23.75	396
Hospice, retirement home	66	9.95	132	19.83	33	4.96	207	31.10	3	0.51	224	33.65	666
Public place, street or other	12	18.90	8	12.43	0	0.00	20	31.08	1	1.85	23	35.74	64
*p value*		*0*.*0037*		*0*.*0124*		*0*.*0181*		<*0*.*0001*		*0*.*1017*		<*0*.*0001*	
**Cause of death**													
Cancer	69	5.57	171	13.76	50	4.02	643	51.73	21	1.68	289	23.25	1243
Cardio-vascular disease	208	24.92	129	15.46	42	5.03	175	20.97	4	0.44	277	33.19	835
Neurological disease	68	11.02	146	23.59	42	6.79	183	29.57	5	0.76	175	28.27	619
Infectious disease	63	21.01	64	21.43	15	5.02	101	33.81	3	0.99	53	17.74	299
Respiratory disease	45	17.29	76	29.37	11	4.25	61	23.57	3	1.17	63	24.35	259
Digestive disease	42	25.77	27	16.53	7	4.29	47	28.78	1	0.75	39	23.88	163
Mental or psychiatric disorder	7	5.57	24	19.85	10	8.27	38	31.43	1	0.97	41	33.91	121
Other cause	64	19.28	45	13.55	21	6.33	64	19.28	0	0.00	138	41.57	332
.	11	20.26	5	9.49	2	3.80	14	26.58	0	0.00	21	39.87	53
*p value*		<*0*.*0001*		<*0*.*0001*		*0*.*1918*		<*0*.*0001*		(*0*.*0728*)		<*0*.*0001*	
**Incurable disease**													
Yes	235	9.63	434	17.79	120	4.92	982	40.25	29	1.17	640	26.23	2440
No	303	23.09	224	17.06	72	5.48	319	24.29	9	0.68	386	29.39	1313
.	38	21.92	31	17.78	7	4.05	26	14.82	0	0.00	72	41.43	173
*p value*		<*0*.*0001*		*0*.*5357*		*0*.*5044*		<*0*.*0001*		*0*.*2138*		*0*.*0231*	

All percentages are weighted for non-response, to ensure there are representative of the initial. The percentage cannot be derived from the absolute unweighted absolute numbers. The p values are obtained with a Fisher exact test. Due to some cells with small numbers, some assumptions of a chi2 test are complied with.

The medical decision to withhold a treatment increases steadily with age from 9.4% with the youngest patients to 22% with the oldest. Conversely, implementation of life-prolonging treatments declines with age, from 24.8% to 11.3%. For patients aged over 50, intensification of alleviation of symptoms varies little with age.

The frequencies of the different decisions by respondent physicians' characteristics are shown in Table 3. The patient’s pathology and the number of deaths certified in the previous three months were directly linked to physician’s speciality: 46% of cancer specialists certified more than 10 deaths in the previous three months compared with 10% of general practitioners, cardiologists and surgeons. Logistic regressions (detailed results not shown) confirm and clarify the decisive influence of the patient’s pathology on the end-of-life decisions made. All other variables held constant, physicians involved in the care of cancer patients reported significantly less often that they had “done everything possible to prolong life”. They more often withheld treatment than those treating other pathologies (except cardio-vascular and digestive diseases), and more often intensified the alleviation of symptoms. The age of the patient does not seem to be a determinant for the type of end-of-life decision (non-treatment decisions or intensifying the alleviation of symptoms). However, the older the patients, the less frequent the decision to do everything possible to prolong life. Lastly, there is little differentiation by physician’s speciality, except for anaesthesiologists and intensivists and emergency physicians, who were more likely to do everything possible to prolong life, and cancer specialists who were more likely to intensify the alleviation of symptoms. Young physicians seem to be less likely to do everything possible to prolong life and are more likely to intensify the alleviation of symptoms. End-of-life decisions are more likely to be made in hospital than at home.

**Table 3:** Frequency of all the different medical end-of-life decisions in France by physicians’ characteristics (non sudden deaths)

	**Life prolonging treatment**		**Withholding treatment**		**Withdrawing treatment**		**Intensification of alleviation of symptoms**		**Administration of a medication to deliberately hasten death**		**No decision**		**Total deaths**
	**n**	**%**	**n**	**%**	**n**	**%**	**n**	**%**	**n**	**%**	**n**	**%**	
**Gender**													
Man	414	15.9	452	17.4	123	4.7	822	31.6	29	1.1	758	29.2	2598
Woman	156	12.1	232	18.0	74	5.8	485	37.7	8	0.6	332	25.8	1287
.	6	16.2	4	10.2	2	5.1	20	50.8	0	0.0	7	17.8	39
*P value*		*0*.*0074*		*0*.*4357*		*0*.*4072*		<*0*.*0001*		*0*.*2600*		*0*.*0453*	
**Age**													
Under 40	120	13.8	161	18.4	50	5.7	351	40.2	14	1.5	178	20.4	874
40-49	168	14.9	198	17.6	75	6.7	371	33.0	3	0.3	310	27.5	1125
50-59	200	14.7	237	17.5	56	4.1	424	31.3	14	1.0	424	31.3	1355
60 and over	86	15.8	87	16.0	16	2.9	169	31.1	7	1.2	179	33.0	543
.	3	9.6	5	18.1	2	7.2	11	39.8	0	0.0	7	25.3	28
*P value*		*0*.*8056*		*0*.*8511*		*0*.*0049*		*0*.*0002*		*0*.*0600*		<*0*.*0001*	
**Speciality**													
General practice	142	11.0	229	17.7	47	3.6	410	31.7	13	1.0	450	34.8	1292
Intensive care	136	29.1	58	12.4	58	12.4	147	31.4	5	1.0	64	13.7	468
Oncology	13	7.1	17	9.5	6	3.4	96	53.9	6	3.1	41	23.0	178
Cardiology	32	26.7	26	21.6	1	0.8	34	28.2	5	4.4	22	18.3	120
Surgery	16	24.1	7	10.8	2	3.1	22	34.1	0	0.0	18	27.9	65
Geriatrics	75	8.8	151	17.7	44	5.2	330	38.7	3	0.3	250	29.3	853
Emergency	81	24.4	63	18.9	15	4.5	50	15.0	0	0.0	124	37.2	333
Neurology	2	4.9	9	24.6	1	2.5	16	43.5	0	0.0	9	24.5	37
Other	78	13.9	125	22.2	25	4.4	213	37.9	6	1.0	115	20.5	562
.	1	4.2	3	14.4	2	9.6	11	52.7	0	0.0	4	19.2	21
*P value*		<*0*.*0001*		*0*.*0014*		(<*0*.*0001*)		<*0*.*0001*		*0*.*0003*		<*0*.*0001*	
**Work context**													
General practice	93	12.2	135	17.7	21	2.8	201	26.4	10	1.3	301	39.5	761
Public hospital	352	17.9	357	18.1	123	6.2	670	34.0	17	0.8	451	22.9	1970
Private hospital	47	16.6	31	11.0	17	6.0	120	42.4	4	1.4	64	22.6	283
Medico-social, rehab. or LTC establishment	16	6.9	51	21.7	7	3.0	99	42.1	0	0.0	62	26.4	235
Other	20	9.2	30	13.6	11	5.0	78	35.4	2	1.0	79	35.8	221
Several work contexts	44	10.6	77	18.7	19	4.6	138	33.5	4	1.1	130	31.5	412
.	4	9.1	6	14.3	1	2.4	21	50.2	0	0.0	10	23.9	42
*P value*		<*0*.*0001*		*0*.*0271*		*0*.*0544*		<*0*.*0001*		(*0*.*5054*)		<*0*.*0001*	
**Size of agglomeration**													
<5000	55	8.5	118	18.4	25	3.9	212	33.0	10	1.6	222	34.6	642
5-10000	60	13.8	74	17.1	16	3.7	130	30.0	3	0.8	150	34.7	433
20-20000	72	15.2	84	17.8	20	4.2	153	32.4	5	1.1	138	29.2	472
20-100000	212	17.7	210	17.5	64	5.3	406	33.9	9	0.8	296	24.7	1197
100-200000	56	14.1	75	18.7	29	7.2	130	32.4	2	0.5	109	27.1	402
>200000	107	16.0	110	16.4	41	6.1	256	38.2	6	0.9	150	22.4	670
.	15	13.7	18	16.5	4	3.7	40	36.6	1	1.2	31	28.4	109
*P value*		<*0*.*0001*		*0*.*9308*		*0*.*0891*		*0*.*1108*		(*0*.*6023*)		<*0*.*0001*	
**Number of deaths certified in the past 3 months**													
0	34	16.8	40	19.5	10	4.9	57	27.8	2	1.1	61	29.8	205
1--2	123	15.0	144	17.6	24	2.9	241	29.4	10	1.2	277	33.8	819
3--4	169	16.0	199	18.8	62	5.9	337	31.9	9	0.9	280	26.5	1056
5--9	149	15.2	156	15.8	50	5.1	354	35.9	8	0.8	269	27.3	986
10--19	60	11.6	100	19.3	34	6.6	202	39.1	5	1.0	116	22.4	517
20+	18	8.5	28	13.2	15	7.1	94	44.3	0	0.0	57	26.9	212
.	22	17.5	21	16.5	5	3.9	39	30.6	3	2.4	37	29.1	127
*P value*		*0*.*0534*		*0*.*2641*		*0*.*0259*		<*0*.*0001*		(*0*.*4672*)		*0*.*0006*	
**In**-**service training on end**-**of**-**life**													
Yes	197	10.6	311	16.7	101	5.4	712	38.3	10	0.6	526	28.3	1858
No	370	18.7	364	18.3	94	4.7	588	29.6	25	1.2	543	27.4	1984
.	8	10.0	13	15.5	5	5.9	26	30.9	3	3.1	29	34.5	84

All percentages are weighted for non-response, to ensure there are representative of the initial. The percentage cannot be derived from the absolute unweighted absolute numbers. The p values are obtained with a Fisher exact test. Due to some cells with small numbers, some assumptions of a chi2 test are complied with.

### Characteristics of the decision-making process

We have exploitable information about how and why the decision was made only for cases where the end-of-life decision and life-prolonging treatment matches the last affirmative answer to questions (1) to (5), i.e. in 91% of cases.

When such a decision was made, 1,706 persons were judged not competent (66% of all decisions) and in 13% of case we had no information about the persons’ competence. We considered that the remaining 545 persons were competent. (21%)

In 70% of the cases, when an end-of-life decision was made, the persons, when competent, were involved in the discussion. The greater the likelihood that the decision made by the physician would hasten death, the more frequently he/she discussed it with the patient, if competent (see Table 4).

**Table 4:** Characteristics of decision-making by type of medical decision (non sudden deaths)

	**Life prolonging treatment**		**Withholding treatment**		**Withdrawing treatment**		**Intensification of alleviation of symptoms**		**Administration of a medication to deliberately hasten death**	
	**n**	%	**n**	%	**n**	%	**n**	%	**n**	%
**Decision discussed with patient** (**by respondent or other physician**)										
Yes	55	9.6	40	8.02	12	8.63	263	19.82	6	16.18
No	79	13.7	40	8.02	3	2.16	44	3.32	2	6.31
Patient not competent	308	53.6	357	71.54	112	80.55	904	68.12	25	67.40
Don't know	19	3.3	6	1.20	3	2.19	26	1.96	1	3.46
No response	114	19.8	56	11.22	9	6.47	90	6.78	2	6.65
Discussion, per 100 competent patients		41.0		50.0		80.0		85.7		71.9
**Decision following patient**'**s explicit request**										
Yes and request repeated	14	2.43	20	4.03	9	6.39	166	12.50	8	21.00
Yes and request not repeated	4	0.69	5	1.01	1	0.62	41	3.09	2	5.85
Yes and do not know whether request repeated	7	1.22	7	1.41	0	0.00	40	3.01	1	2.63
No	418	72.57	392	79.03	117	83.06	962	72.44	22	58.92
No response	133	23.09	72	14.52	14	9.94	119	8.96	4	11.60
**Patient expressed wish at some moment to hasten death**										
Yes	13	2.26	46	9.26	14	10.00	203	15.31	14	38.35
No	333	57.81	261	52.52	86	61.43	809	61.01	11	28.99
Do not know	115	19.97	126	25.35	30	21.43	210	15.84	10	27.30
No response	115	19.97	64	12.88	10	7.14	104	7.84	2	5.36
**Decision discussed with another person**										
**incompetent person**										
Doctors	102	33.12	136	38.10	62	55.36	434	48.01	14	56.00
Nurses and nursing assistants, medical care team	49	15.91	95	26.61	42	37.50	326	36.06	10	40.00
Family	74	24.03	171	47.90	64	57.14	549	60.73	19	76.00
Other people	1	0.32	3	0.84	0	0.00	4	0.44	0	0.00
Trusted third party	19	6.17	29	8.12	15	13.39	150	16.59	5	20.00
As part of a collective decision	16	5.19	24	6.72	15	13.39	137	15.15	5	20.00
No	119	38.64	48	13.45	4	3.57	57	6.31	1	4.00
No response	7	2.27	8	2.24	1	0.89	11	1.22	0	0.00
**Trusted third party**										
**incompetent person**										
Yes and involved in discussion phase	57	18.51	71	19.89	22	19.64	312	34.55	8	32.00
Yes and not involved in discussion phase	19	6.17	7	1.96	6	5.36	11	1.22	1	4.00
Yes and do not know whether involved or not	4	1.30	2	0.56	1	0.89	3	0.33	0	0.00
No	99	32.14	108	30.25	31	27.68	295	32.67	10	40.00
No because patient not capable of naming one	54	17.53	105	29.41	27	24.11	177	19.60	4	16.00
Do not know	75	24.35	64	17.93	25	22.32	105	11.63	2	8.00
**Advance directive**										
**incompetent person**										
Yes and was an important factor in the decision	1	0.32	2	0.56	0	0.00	9	1.00	2	8.00
Yes and was not important factor in the decision	0	0.00	2	0.56	0	0.00	3	0.33	0	0.00
Yes and do not know whether important factor in the decision	0	0.00	0	0.00	0	0.00	0	0.00	0	0.00
No	185	60.06	233	65.45	75	66.96	685	75.86	16	64.00
Do not know	122	39.61	119	33.43	37	33.04	206	22.81	7	28.00
**Best description of the medical act**										
Symptom treatment	337	58.41	144	28.92	17	12.15	717	54.03	7	19.44
Decision to withhold or withdraw treatment	20	3.47	237	47.59	104	74.31	123	9.27	6	17.06
Sedation for distress in terminal phase	10	1.73	19	3.82	1	0.60	361	27.21	17	45.59
Euthanasia	0	0.00	0	0.00	1	0.79	1	0.07	5	12.04
Medically assisted suicide	0	0.00	0	0.00	0	0.00	0	0.00	0	0.00
Other	66	11.44	30	6.02	6	4.29	18	1.36	0	0.00
No response	144	24.96	68	13.65	11	7.86	107	8.06	2	5.87

All percentages are weighted for non-response, to ensure there are representative of the initial. The percentage cannot be derived from the absolute unweighted absolute numbers.

According to the responding physicians, when an end-of-life decision or an explicit life-prolonging decision was made, 16% of persons had expressed at some point a wish to hasten death, although only 1.7% had explicitly requested euthanasia. The decision was made at the patient's explicit request in almost 15% of cases. The greater the likelihood that the decision would hasten death, the higher the percentage of persons who had expressed a wish to hasten death (from 8% for those with a treatment withheld to 38% for those with a medication given to deliberately hasten death) or who requested euthanasia (0.5 to 17%).

When an end of life decision or an explicit life-prolonging decision was made and when the patient was incompetent, 1.5% of the persons had expressed their wishes through written advance directives. For the responding physicians, these advance directives were an important part of the decision in 72% of cases. 50% of patients had appointed a trusted third party, who took part in discussions about decisions to be made at later stages of the disease in 90% of cases. The decisions were discussed in 45% of cases with colleagues and in 31% of cases with nursing staff members. No such discussion (either with colleagues and/or nursing staff, and/or described as a part of a “collective" process) was reported in 14% of cases. These figures varied according to the type of decision: discussions with colleagues, family, or trusted third party were more frequent when decisions were more likely to hasten death (Table 4).

When a drug was administered to deliberately hasten death on the patient’s explicit request, this request was repeated 8 times out of 11, and an explicit request for euthanasia was made in 6 cases. When the sole decision was to prolong life, it was at patient’s explicit request in 4% of the cases.

Among the 55 explicit requests for euthanasia reported by the physicians, 6 were granted, whereas in 44 cases the physician chose to intensify the alleviation of symptoms, and in 1 case no decision was reported, except for doing everything possible to prolong the life.

For almost half of the physicians, “deep sedation for distress in terminal phase” was the term that best described the decision to deliberately administer a medication to hasten death; much less frequently “symptom treatment” or “non-treatment decision”. Only 5 physicians reported “euthanasia”.

## Discussion and conclusions

### Main findings

For the first time, this study provides data on end-of-life medical decisions on a representative sample of all deaths in France.

In 12.2% of cases, the decision was to do everything possible to prolong life. Non-treatment decisions were made in 16.8% of cases, treatment was withheld in 14.6% and withdrawn in 4.2%. Alleviation of symptoms with opioids and/or benzodiazepines was intensified in 28.1% of cases, A drug was administered to deliberately hasten death in 0.8% of cases, at the patient's request in 11 out of the 38 cases concerned.

The study shows that end-of-life medical decisions that may hasten death are relatively frequent in France. Most of such decisions are in compliance with the law, which allows physicians to withhold or withdraw life prolonging treatment and to intensify alleviation of symptoms even if unintended side effects may hasten death (“double effect”), as long as the first intention is not to hasten death. In a much smaller number of cases (3.1%), the death followed a decision made with the declared intention of hastening death. The patient's pathology is the main factor governing this type of decision. Even though most end-of-life medical decisions are made in compliance with the 2005 law, and decisions leading to a strong likelihood of death are more frequently taken after discussion with the patient or trusted third party and the medical staff (other doctors, nursing staff), the study shows that the legal provisions governing these decisions are not always fully respected.

### Strengths and limitations

For the first time in France, this study provides data on end-of-life decisions on a representative sample of deaths, whatever the cause, wherever the death took place. It gives objective results on this important issue that will inform and assist both public and legislative debate. The French national end-of-life watchdog *Observatoire National de la Fin de Vie* (ONFV) has noted the lack of available scientific data on medical practices in this regard in France [13].

This survey also shows that investigating this sensitive topic and even exploring illegal practices is possible in France; this was by no means certain when the study was first launched. That is why we deliberately adapted the questionnaire to take account of the French legislative context and of the French sensitivity on this issue revealed in preliminary tests, even at the expense of comparability with other countries.

From a methodological viewpoint, we conducted the survey using a mixed mode approach, i.e. enabling physicians to answer either by Internet or by returning the questionnaire in a paid-reply envelope. We developed a different process for each of the response channels in order to guarantee total anonymity.

The 40% participation rate is towards the bottom of the range of the European EURELD surveys and could be considered as a limitation [4]. One explanation for this low rate might be that, unlike some of the EURELD surveys with a higher response rate, we did not stratify the sample according to the likelihood that death followed a potential end-of-life decision, and therefore sent our questionnaire to proportionally more physicians who would probably consider their patient's end of life to be irrelevant to the survey. But in fact, this participation rate is fairly close to that for other surveys of French physicians [7,14]. In the non-response survey, the main reasons given were lack of time and refusal to take part in any kind of survey. Few doctors mentioned the survey topic as a reason for not responding. The length of the questionnaire and, above all, the need to look through the patient’s case history may have been dissuasive. Some doctors did not feel the survey concerned them, especially if they had not been treating the patient prior to death, as the under-representation of deaths from external causes also suggests.

Nevertheless, the comparison of respondent and non-respondent physicians’ profiles reveals no significant differences, lending support to our belief that this assessment of end-of-life medical decisions is likely to be reliable, although an under-estimation of illegal practices cannot be excluded.

This survey, like others on the same topic, [2,4,15-27] is based on the responses of physicians, who are best placed to answer questions about decisions for which they have taken responsibility. However, one limitation is that they can only report on their own experience of a reality that also involves other people (the patient, the family or friends, other medical staff etc.) who might have different points of view.

### Comparison with end-of-life decisions in other studies

The only figures available in France about end-of-life decisions concerned withholding or withdrawal of life support were conducted in 2004, prior to the law. In the MAHO survey [28], carried out in public hospitals, withholding or withdrawing life support was less frequent than in our results concerning all public hospitals (45.4% of the deceased patients included in the study, vs. 51.2% in our survey). In the DALISA survey [8,14], carried out in emergency departments, withholding life support was observed in 41.5% of the deaths, and withdrawing (alone or preceded by withholding) life support in 58.5%, vs. 89% and 11% respectively, in our results concerning emergency departments. These differences may be partially explained by the different study designs; the legislation has also changed between these studies and ours.

It is not easy to compare study findings with those conducted elsewhere, even where similar survey protocols are used (retrospective survey of certifying physicians, with a representative sample of deaths). The definition we used for sudden deaths and the wording and approach for questions about end-of-life medical decisions were different to those used in EURELD type studies. In particular, we chose a two-step approach: a question on a treatment/decision (withholding, withdrawing a treatment, intensifying the alleviation of pain); and, for each decision made, its possible or certain effect on hastening death as well as the intention of hastening death, is investigated. According to Seale [29] who compared the two wordings for UK, the two-step approach gives a lower percentage of end-of-life decisions compared to the approach where the potential effect of hastening death is included in the question of treatment. As a result, end-of-life decisions could not be classified in an identical manner as Eureld, although we tried to get as close as possible. If we had replaced our definition of “sudden death” (deaths declared by physician as “sudden and unexpected” and for which they cannot provide any information about the patient's end of life) with the EURELD definition, the percentage of sudden deaths would have more than doubled (from 16.9 to 39.3%). This change of definition of sudden deaths reduces more the proportion of medical decisions without any intention regarding deaths than the one that possibly or certainly hastened deaths.

Compared with the 2001 Eureld survey results [4] and more recent results in Belgium [30] and the Netherlands [31], and taking the definitions of medical decisions closest to those used in these surveys, the proportion of sudden deaths in the French data (39%) is higher than that of all other countries (29-34%). The percentage of deaths for which a decision was made that possibly or certainly hastened death (40%), is around the average observed in the other European countries. It is much higher than in Italy, (29% in 2001), but well below the levels in Switzerland, where assisted suicide is legal (51% in 2001), and in the Netherlands (57% in 2010) and Belgium (48% in 2007), two countries where euthanasia has been legalised. In France, intensification of treatment to alleviate pain and/or symptoms is close to the level observed in Belgium in 2007, but slightly higher than in most EURELD countries. However, levels of withholding or withdrawal of treatment are similar to those observed in more recent surveys in Belgium and the Netherlands.

France ranks among the countries with a low percentage of physician-assisted dying by administration of a drug to deliberately hasten death. At less than 1%, this level is close to the Danish level in 2001, it is higher than in Sweden and Italy and much lower than in the Netherlands and Belgium. No physician-assisted suicide was reported and euthanasia (at the patient’s request) is very rare. According to our results, a fifth of medical decisions that possibly or certainly hastened deaths are made at the patient’s request, (a third for deaths with a decision to administer a medication to deliberately hasten death). This is much lower than in the Netherlands and Belgium (where Euthanasia is legal). It is higher than in other European countries in the 2001 Eureld survey. Discussion of the decision with competent patients was more frequent in France (80%) than in most European countries in 2001 with the exception of the Netherlands. Also for non-competent patients, the family is very often involved in the discussion (78%), less frequently than in the Netherlands, similarly to Belgium-Switzerland but much more frequently than in other countries. This might reflect an effect of the French law on discussion with patients or relatives.

Overall, the main results on end-of-life medical decisions are consistent with those of surveys conducted in other countries: intensification of pain relief treatment is the most common decision [17] and administration of drugs to intentionally end the patient’s life is rare.

### Discussion of the findings in light of the French law

In France, the 2005 law on patients’ rights and the end of life defined a legal framework allowing patients to refuse any treatment they consider unreasonable, and allowing doctors to decide on treatments that may have the side effect of hastening death, in accordance with the wishes expressed by the patient [1]. The medical decisions observed in our survey mostly complied with French legal requirements, as the 2005 Act allows withholding and withdrawal of life support, and intensified alleviation of symptoms even when it may (unintentionally) hasten death. Indeed 80% of the physicians who made this decision said they were aware of its potential “double effect”. Some decisions overstepped the law, although very rarely. A drug was administered with the explicit intention of hastening death – an act that can be considered as poisoning under French law – at the patient's explicit request in 0.2% of these deaths, and without a clear patient request in another 0.6%. Intention to hasten death was also declared, even if very infrequently, in some of the decisions of life support withholding or withdrawal or of intensified alleviation of symptoms. As a whole, decisions with intention to hasten death amounted to 3.1% of all deaths, and only one out five of these decisions was made on the patient's explicit request, whereas such a request is mandatory in all countries where the law permits euthanasia in specific cases, and is part of the ONFV definition of euthanasia [1].

The decision making processes observed in our survey were far from complying with the 2005 legal procedures, which are required whatever the end-of-life decision made. A discussion with the patient when competent was mentioned by the physician in only 72% of the cases when a drug was administered intentionally to hasten death, in 80% when life support was withdrawn, and 41% when everything was done to prolong life.

When an end-of-life decision is made for an incompetent patient, advance directives if any, discussion with a trusted third party previously named by the patient, if any, discussion with the family, if any, discussion with a colleague not in charge of the patient, with colleagues and with nursing staff members, are compulsory components of the decision-making process. When a treatment was withdrawn for a possibly incompetent patient, the decision was discussed with other doctors in 39% of cases, with the nursing staff in 27% of cases and with the family in 50% of cases. The physician made this decision alone in 14% of cases. When a drug was administered with the intention of hastening death, the decision was discussed in 14, 10, 19 and 4 cases out of 24, respectively.

Looking at these discrepancies between legal requirements and actual practice, we should not forget that our survey concerned deaths that occurred in December 2009, less than three years after the revision of the medical ethics charter. There is still a lot to be done through medical education and population awareness-raising to ensure that no physician is obliged to face such difficult decisions alone.

## Conclusion

In conclusion, these results provide an overview of end-of-life medical decisions in France, three years after the 2005 regulations were enacted, and for the first time on a large sample representative of all kinds of deaths. They are objective results in the context of the current legislation. They will help medical authorities and policy makers to examine how the act of parliament is applied and to understand more clearly which features of the current law are difficult to comply with. They will inform and assist the current public debate on this important topic. They will also serve as a baseline to investigate future changes.

## Competing interests

The authors declare that they have no competing interest.

## Authors’ contributions

SP participated in the conception and design of the survey and study, supervised the data collection, coordinated the study, performed the statistical analyses and drafted the manuscript. AM participated in the conception and design of the survey and study, supervised the data collection, performed the statistical analysis and draft the manuscript. SP and RA participated in the conception and design of the survey and study, critically revised the manuscript for important content. All authors read and approved the final manuscript.

## Pre-publication history

The pre-publication history for this paper can be accessed here:

http://www.biomedcentral.com/1472-684X/11/25/prepub

## Supplementary Material

Additional file 1 Key questions on medical decisions of end-of-life in the French survey. Click here for file

Additional file 2 Questionnaire of the French survey on End-of-life. Click here for file
