# Supplementary material for: End-of-life medical decisions in France: a death certificate follow-up survey 5 years after the 2005 act of parliament on patients’ rights and end of life
Source: BMC Palliat Care. 2012 Dec 3;11:25. doi: 10.1186/1472-684X-11-25 (PMC3543844; doi:10.1186/1472-684X-11-25)
Supplement: Additional file 1 — Key questions on medical decisions of end-of-life in the French survey. [file 1472-684X-11-25-S1.pdf]

Pour finir, quelques questions sur la participation aux enquêtes en général

37 De façon générale, répondez-vous aux enquêtes qui vous sont adressées ?

- ❑ La plupart du temps
- ❑ Souvent
- ❑ De temps en temps
- ❑ Rarement
- ❑ Jamais

37.1 Qu'est-ce qui vous incite plutôt à répondre à une enquête ?

- ❑ Le thème de l'enquête
- ❑ Le temps dont vous disposez
- ❑ Le promoteur de l'enquête

37.2 Répondez-vous plutôt aux enquêtes émanant ...

- ❑ d'une autorité de santé
- ❑ d'une société savante
- ❑ d'une institution de recherche
- ❑ d'un laboratoire pharmaceutique
- ❑ Autre

38 Le thème de l'enquête vous a-t-il...

- ❑ intéressé
- ❑ gêné
- ❑ laissé indifférent
- ❑ sans opinion

39 Avez-vous au titre de la formation médicale continue, suivi une formation sur la fin de vie ?

- ❑ Oui
- ❑ Non

40 Pensez-vous qu'une enquête comme celle-ci puisse...
(Plusieurs réponses possibles)

- ❑ sensibiliser le corps médical
- ❑ améliorer la prise en charge de la fin de vie
- ❑ nourrir le débat sur la fin de vie
- ❑ aider le législateur
- ❑ n'avoir aucun de ces effets
- ❑ je ne me prononce pas

Merci de votre collaboration

OBSERVATOIRE NATIONAL DE LA FIN DE VIE

INED
Institut national d'études démographiques

ENQUÊTE FDV

Enquête
La fin de vie en France

2010

INED, 133, boulevard Davout
75980 Paris Cedex 20

tél. : 01.56.06.43.00
email : enquetefindevie@ined.fr
site web : http://fdv.site.ined.fr

CépiDc Inserm

Liberté • Égalité • Fraternité
RÉPUBLIQUE FRANÇAISE
MINISTÈRE DE LA SANTÉ ET DES SPORTS

ORDRE NATIONAL DES MEDECINS
Conseil National de l'Ordre

Commençons d'abord par quelques questions vous concernant...

1.1 Vous êtes...

❑ un homme ❑ une femme

1.2 Quel est votre âge ?

❑ Moins de 40 ans
❑ 40 à 49 ans
❑ 50 à 59 ans
❑ 60 ans ou plus

1.3 Vous exercez...

❑ la médecine générale
❑ l'anesthésie réanimation, la réanimation médicale
❑ la cancérologie/oncologie
❑ la cardiologie
❑ la chirurgie
❑ la gériatrie
❑ la médecine d'urgence
❑ la neurologie
❑ la pédiatrie
❑ une autre spécialité

1.4 Dans quel cadre ?
(Plusieurs réponses possibles)

❑ Dans un cabinet libéral
❑ Dans un hôpital public
❑ Dans une clinique privée
❑ Dans un établissement médico-social
❑ Dans un établissement de soins de suite
❑ Dans une unité de soins de longue durée (USLD)
❑ Autre

1.5 Combien y a-t-il d'habitants dans la commune où vous exercez ?

❑ Moins de 5 000 habitants
❑ De 5 000 à moins de 10 000 habitants
❑ De 10 000 à moins de 20 000 habitants
❑ De 20 000 à moins de 100 000 habitants
❑ De 100 000 à moins de 200 000 habitants
❑ Plus de 200 000 habitants

1.6 Combien de décès avez-vous certifiés durant les trois derniers mois ?

❑ 0
❑ 1 à 2
❑ 3 à 4
❑ 5 à 9
❑ 10 à 19
❑ 20 et plus

33 Dans la semaine précédant sa mort, la personne décédée a-t-elle reçu des visites ?

❑ Oui → 33.1
❑ Non
❑ Je ne sais pas

33.1 S'agissait-il...

❑ seulement de membres de sa famille
❑ de membres de sa famille et d'amis
❑ je ne sais pas s'il s'agissait d'amis ou de membres de la famille

34 A votre avis, les conditions de ce décès ont-elles été, dans l'ensemble, conformes aux attentes du patient ?

❑ Oui
❑ Non
❑ Je ne me prononce pas

35 Aujourd'hui, pensez-vous que cette fin de vie s'est déroulée dans des conditions...

❑ très correctes
❑ plutôt correctes
❑ plutôt mauvaises
❑ très mauvaises
❑ je ne me prononce pas

36 Dans quelle mesure ce décès vous a-t-il éprouvé ?

0 ❑ 1 ❑ 2 ❑ 3 ❑ 4 ❑ 5 ❑ 6 ❑ 7 ❑ 8 ❑ 9 ❑ 10 ❑
(pas du tout) *(beaucoup)*

32 Le patient avait-il fait une demande explicite d'euthanasie ?

- ❑ Oui
- ❑ Non

32.1 Pour quel(s) motif(s) ?
(Plusieurs réponses possibles)

- ❑ Les douleurs ou les symptômes étaient réfractaires à la thérapeutique
- ❑ Le patient ressentait une angoisse de mort persistante
- ❑ Le patient éprouvait un sentiment d'indignité
- ❑ Autre

32.2 A-t-on accédé à cette demande ?

- ❑ Oui
- ❑ Non

32.3 Pour quelle(s) raison(s) n'a-t-on pas accédé à cette demande ?
(Plusieurs réponses possibles)

- ❑ Le patient ne se trouvait pas au stade terminal
- ❑ Il y avait encore une perspective médicale pour atténuer la souffrance ou les symptômes
- ❑ La demande n'était pas mûrement réfléchie
- ❑ La demande était influencée par un tiers
- ❑ En raison d'objections de principe à l'euthanasie
- ❑ Le patient a renoncé à sa demande
- ❑ Le(la) conjoint(e) et/ou la famille a demandé de ne pas répondre à cette demande
- ❑ Par peur des conséquences judiciaires
- ❑ Pour respecter la loi
- ❑ Autre raison

Maintenant, quelques questions concernant la personne décédée

2.1 Sexe :

- ❑ un homme
- ❑ une femme

2.2 Groupe d'âge :

- ❑ Moins de 18 ans
- ❑ 18 à 29 ans
- ❑ 30 à 49 ans
- ❑ 50 à 69 ans
- ❑ 70 à 79 ans
- ❑ 80 à 89 ans
- ❑ 90 ans et plus

2.3 Mois du décès :

- ❑ Janvier
- ❑ Février
- ❑ Mars
- ❑ Avril
- ❑ Mai
- ❑ Juin
- ❑ Juillet
- ❑ Août
- ❑ Septembre
- ❑ Octobre
- ❑ Novembre
- ❑ Décembre

2.4 Profession de la personne décédée ?
(Si elle était retraitée, indiquer la profession avant la retraite)

- ❑ Agriculteur
- ❑ Artisan, commerçant, chef d'entreprise
- ❑ Cadre, profession intellectuelle supérieure *(professeur...)*
- ❑ Profession intermédiaire *(instituteur, infirmier...)*
- ❑ Employé
- ❑ Ouvrier
- ❑ Retraité *(si aucune idée de la profession exercée avant la retraite)*
- ❑ Sans profession
- ❑ Autre
- ❑ Je ne sais pas

2.5 Région du décès :

- ❑ Alsace
- ❑ Aquitaine
- ❑ Auvergne
- ❑ Basse-Normandie
- ❑ Bourgogne
- ❑ Bretagne
- ❑ Centre
- ❑ Champagne-Ardenne
- ❑ Corse
- ❑ Franche-Comté
- ❑ Haute-Normandie
- ❑ Île-de-France
- ❑ Languedoc-Roussillon
- ❑ Limousin
- ❑ Lorraine
- ❑ Midi-Pyrénées
- ❑ Nord-Pas de Calais
- ❑ Pays de la Loire
- ❑ Picardie
- ❑ Poitou-Charentes
- ❑ Provence-Alpes-Côte d'Azur
- ❑ Rhône-Alpes

2.6 Lieu du décès :

- ❑ Logement privé, domicile
- ❑ Établissement hospitalier
- ❑ Clinique privée
- ❑ Hospice, maison de retraite
- ❑ Voie ou lieu public
- ❑ Autre lieu

2.7 Cause initiale du décès :

- ❑ Cancer
- ❑ Maladie cardio-vasculaire
- ❑ Maladie neurologique, cérébro-vasculaire
- ❑ Maladie infectieuse
- ❑ Maladie de l'appareil respiratoire *(hors cancer)*
- ❑ Maladie de l'appareil digestif *(hors cancer)*
- ❑ Troubles mentaux et psychiatriques
- ❑ Mort violente
- ❑ Autres causes

3 À quand remonte votre premier contact avec la personne décédée ?

- ❑ Après le décès
- ❑ Avant ou au moment du décès →

3.1 S'agissait-il d'un patient dont vous aviez la charge ?

- ❑ Oui
- ❑ Non

4 Le patient était-il suivi pour une pathologie incurable ?

- ❑ Oui
- ❑ Non
- ❑ Je ne sais pas

5 S'agit-il d'un décès soudain et tout à fait inattendu ?

- ❑ Oui →
- ❑ Non

5.1 Pensez-vous pouvoir apporter des informations sur la fin de vie de ce patient ?

- ❑ Oui
- ❑ Non ⇒ **Allez à question 37 page 20**

29 Vous (ou un autre médecin) avez-vous discuté avec quelqu'un d'autre de la décision prise concernant le dernier acte médical cité avant qu'il soit décidé d'y procéder ?
(Plusieurs réponses possibles)

- ❑ Oui, avec un ou plusieurs médecins
- ❑ Oui, avec un ou plusieurs infirmiers ou aides-soignants
- ❑ Oui, avec l'équipe soignante
- ❑ Oui, avec le conjoint et/ou la famille du patient
- ❑ Oui, avec la personne de confiance
- ❑ Oui, avec d'autres personnes
- ❑ Oui, dans le cadre de la procédure collégiale
- ❑ Non
- ❑ Je ne sais pas

29.1 Cette discussion avait-elle pour but...
(Plusieurs réponses possibles)

- ❑ d'avertir
- ❑ de prendre un avis
- ❑ de prendre une décision commune

29.2 Les éléments de la discussion et la décision sont-ils inscrits dans le dossier ?

- ❑ Oui
- ❑ Non
- ❑ Je ne sais pas

30 Qu'est-ce qui a principalement motivé le dernier acte cité ?
(Plusieurs réponses possibles)

- ❑ La mort ne semblait pas inéluctable à ce moment-là
- ❑ Douleur non soulagée du patient malgré le traitement antalgique
- ❑ Symptômes non contrôlables du patient *(asphyxie aiguë, hémorragie cataclysmique...)*
- ❑ Souffrance morale du patient
- ❑ Absence de perspective d'amélioration
- ❑ Souhait de ne pas prolonger inutilement la survie
- ❑ Demande ou souhait du patient
- ❑ Demande ou souhait du conjoint, de la famille ou de la personne de confiance
- ❑ Autre raison

31 Selon vous, quel terme s'applique le mieux au dernier acte cité ?
(Une seule réponse possible)

- ❑ Traitement des symptômes
- ❑ Décision de non-traitement ou d'arrêt de traitement
- ❑ Sédation pour détresse en phase terminale
- ❑ Euthanasie *("acte d'un tiers qui met délibérément fin à la vie d'une personne, dans l'intention de mettre un terme à une situation jugée insupportable" Comité consultatif national d'éthique)*
- ❑ Suicide médicalement assisté *(fourniture par le médecin d'une substance que le patient prend lui-même)*
- ❑ Autre

27 **La décision relative à ce dernier acte cité a-t-elle été prise suite à une demande explicite du patient ?**

- ❑ Oui, suite à une demande orale
- ❑ Oui, suite à une demande écrite
- ❑ Oui, suite à une demande orale et écrite
- ❑ Non

27.1 **Cette demande a-t-elle été réitérée ?**

- ❑ Oui
- ❑ Non
- ❑ Je ne sais pas

28 **Le patient avait-il fait part à un moment ou à un autre d'un souhait d'avancer la survenue de la mort ?**

- ❑ Oui, explicitement
- ❑ Oui, mais pas explicitement
- ❑ Non
- ❑ Je ne sais pas

28.1 **Comment avez-vous été informé de ce souhait ?**

- ❑ Verbalement par le patient
- ❑ Par des directives anticipées écrites
- ❑ Par un autre média *(enregistrement sonore, etc.)*
- ❑ Par un proche du patient
- ❑ Par l'équipe soignante
- ❑ Autrement

Lieu du décès, soins et traitements

6 **Dans quels lieux le patient a-t-il séjourné au moment du décès et au cours des quatre semaines précédentes ?**
Cochez une case par ligne

	Hôpital *(public ou privé, y compris USLD)*	Domicile	Maison de repos/ de retraite/ EHPAD	Autre lieu	Je ne sais pas
Le 28ème jour avant le décès	❑	❑	❑	❑	❑
Le 7ème jour avant le décès	❑	❑	❑	❑	❑
La veille du décès	❑	❑	❑	❑	❑
Le jour du décès	❑	❑	❑	❑	❑

6.1 **Etait-ce dans un service de... ?**

- ❑ Réanimation
- ❑ Urgences
- ❑ Cancérologie/Oncologie
- ❑ Cardiologie
- ❑ Chirurgie
- ❑ Gériatrie
- ❑ Médecine polyvalente
- ❑ Neurologie
- ❑ Soins palliatifs
- ❑ Autre

7 **Connaissez-vous le lieu où le patient souhaitait décéder ?**

- ❑ Oui
- ❑ Non

7.1 **Où souhaitait-il décéder ?**

- ❑ À domicile
- ❑ En maison de repos/retraite/EHPAD
- ❑ À l'hôpital ou en clinique
- ❑ Autre

7.2 **Est-il décédé au lieu souhaité ?**

- ❑ Oui
- ❑ Non

7.3 **Pour quelle(s) raison(s) n'est-il pas décédé au lieu souhaité ?**
(Plusieurs réponses possibles)

- ❑ La mort est survenue plus rapidement que prévu
- ❑ Les proches s'y sont opposés
- ❑ Les soins étaient trop complexes ou trop lourds pour y être effectués
- ❑ Autres raisons

8 **Connaissez-vous le lieu où le conjoint (et/ou la famille) du patient souhaitait qu'il décède ?**

- ❑ Oui
- ❑ Non
- ❑ Le patient n'avait ni conjoint ni famille
- ❑ Je ne sais pas si le patient avait un conjoint ou une famille

8.1 **Quel était le souhait du conjoint (et/ou de la famille) quant au lieu de décès du patient ?**

- ❑ À domicile
- ❑ En maison de repos/retraite/EHPAD
- ❑ À l'hôpital ou en clinique
- ❑ Autre

8.2 **Ce souhait s'est-il réalisé ?**

- ❑ Oui
- ❑ Non

8.3 **Pour quelles raisons, le patient n'est-il pas décédé au lieu souhaité par le conjoint (et/ou la famille) ?**
(Plusieurs réponses possibles)

- ❑ La mort est survenue plus rapidement que prévu
- ❑ Le patient s'y est opposé
- ❑ Les soins étaient trop complexes ou trop lourds pour y être effectués
- ❑ Autres raisons

Le dernier acte médical cité

***ATTENTION** : les questions 25 à 31 concernent <u>le dernier acte cité</u>, c'est-à-dire la <u>dernière</u> réponse "oui" que vous avez donnée aux questions 20 à 24.*

25 **Vous, ou un confrère, avez-vous discuté avec le patient de la décision prise lors du dernier acte cité ?**

- ❑ Oui

25.1 **Au cours de cette discussion, avez-vous trouvé le patient capable d'apprécier sa situation ?**

- ❑ Oui
- ❑ Non
- ❑ Je ne sais pas

25.2 **Au cours de cette discussion, avez-vous trouvé le patient capable de s'autodéterminer ?**

- ❑ Parfaitement
- ❑ Partiellement
- ❑ Pas du tout
- ❑ Je ne me prononce pas

- ❑ Non

26 **Pour quelle(s) raison(s) n'a-t-on pas discuté avec le patient de la décision prise lors du dernier acte cité ?**
(Plusieurs réponses possibles)

- ❑ En tant que médecin, j'estime ne pas avoir à aborder cette question avec un patient
- ❑ En tant que médecin, je ne me sentais pas capable d'aborder cette question
- ❑ En tant que médecin, je ne savais pas quelle serait l'attitude la plus adaptée
- ❑ Le patient était trop jeune
- ❑ Le patient était dans un état peu conscient ou inconscient
- ❑ Le patient était atteint de démence
- ❑ Le patient présentait une déficience mentale
- ❑ Le patient souffrait de troubles psychiatriques
- ❑ Le dernier acte cité était clairement le meilleur pour le patient
- ❑ La discussion aurait fait plus de mal que de bien au patient
- ❑ Le patient se voyait en meilleure santé qu'il n'était
- ❑ Autre raison

- ❑ Je ne sais pas

24 **Concernant cette personne, avez-vous pris la décision d'utiliser un ou des médicaments pour mettre délibérément fin à sa vie ?**

- ❑ Oui
- ❑ Non

24.1 **De quel(s) médicament(s) s'agit-il ?**
(Plusieurs réponses possibles)

- ❑ Barbiturique
- ❑ Benzodiazépine
- ❑ Dolosal- largactil- phénergan (DLP)
- ❑ Morphine ou autre opiacé
- ❑ Neuroleptique
- ❑ Myorelaxant (curare)
- ❑ Potassium
- ❑ Autre médicament

24.2 **Qui a administré ce(s) médicament(s) ?**
(Plusieurs réponses possibles)

- ❑ Vous ou un autre médecin
- ❑ Un(e) infirmier(ère)
- ❑ Le patient lui-même
- ❑ Une autre personne

24.3 **Si le(s) médicament(s) n'a (ont) pas été administré(s) par vous ou par un autre médecin, étiez-vous présent lors de l'administration ?**

- ❑ Oui
- ❑ Non

24.4 **Un autre médecin était-il présent lors de l'administration ?**

- ❑ Oui
- ❑ Non

Si vous n'avez répondu " oui " à aucune des questions 20 à 24 ⇒ **Allez à la question 32**

9 **Le patient avait-il désigné une personne de confiance ?**
(La personne de confiance au sens de la loi du 22 avril 2005 est la personne désignée par le patient comme porte-parole s'il n'a plus la capacité de s'autodéterminer)

- ❑ Oui
- ❑ Non
- ❑ N'avait pas la capacité de le faire *(inconscient,...)*
- ❑ Je ne sais pas

9.1 **S'agissait-il ?**

- ❑ Du conjoint
- ❑ Du père ou de la mère
- ❑ D'un frère ou d'une sœur
- ❑ D'un de ses enfants ou descendants
- ❑ D'un autre membre de la famille
- ❑ D'un(e) ami (e)
- ❑ Du médecin traitant
- ❑ D'une autre personne

9.2 **La personne de confiance a-t-elle été associée aux discussions concernant les décisions à prendre en phase terminale ?**

- ❑ Oui
- ❑ Non
- ❑ Je ne sais pas

9.3 **Une autre personne de l'entourage a-t-elle été associée aux discussions concernant les décisions à prendre en phase terminale ?**

- ❑ Oui
- ❑ Non
- ❑ Je ne sais pas

10 **Le patient avait-il rédigé des directives anticipées ?**
(Une directive anticipée est un écrit rédigé par une personne majeure par lequel elle fait connaître ses désirs quant aux questions relatives à sa fin de vie, en particulier sur la question de l'arrêt ou de la limitation des traitements)

- ❑ Oui
- ❑ Non
- ❑ Je ne sais pas

10.1 **Les directives anticipées ont-elles constitué un élément important dans les décisions en phase terminale ?**

- ❑ Oui
- ❑ Non
- ❑ Je ne sais pas

11 **Pour chacun des intervenants suivants, cochez la case correspondant à leur implication dans la prise en charge du patient au cours du dernier mois avant le décès :**
(Cochez une case par ligne)

	Impliqué	Pas impliqué	Je ne sais pas
Médecin traitant	❑	❑	❑
Médecin spécialiste dans la prise en charge de la douleur	❑	❑	❑
Médecin spécialiste en soins palliatifs	❑	❑	❑
Médecin d'une autre spécialité	❑	❑	❑
Psychiatre	❑	❑	❑
Réseau de soins palliatifs	❑	❑	❑
Infirmier	❑	❑	❑
Kinésithérapeute	❑	❑	❑
Aide-soignant	❑	❑	❑
Psychologue	❑	❑	❑
Assistante sociale	❑	❑	❑
Accompagnant bénévole	❑	❑	❑
Représentant d'une religion *(aumônier, pasteur, rabbin, imam, etc.)*	❑	❑	❑
Famille, amis	❑	❑	❑
Autre	❑	❑	❑

12 **À quoi étaient principalement destinés les traitements au cours de la dernière semaine avant le décès ?**

- ❑ La guérison
- ❑ Le traitement d'un épisode aigu émaillant une affection chronique
- ❑ Le traitement de plusieurs épisodes aigus émaillant une affection chronique
- ❑ Le seul confort du patient

13 **Selon vous, dans quelle mesure (de 0 à 10) les symptômes suivants ont-ils été rencontrés chez le patient au cours des dernières 24 heures avant le décès (en dépit d'un éventuel traitement) ?**
(Mettez une croix dans la colonne correspondant à votre appréciation)

	0	1	2	3	4	5	6	7	8	9	10	
Absence de douleur	❑	❑	❑	❑	❑	❑	❑	❑	❑	❑	❑	Douleur maximale imaginable
Pas de nausée	❑	❑	❑	❑	❑	❑	❑	❑	❑	❑	❑	Nausée sévère
Pas de troubles digestifs	❑	❑	❑	❑	❑	❑	❑	❑	❑	❑	❑	Troubles digestifs sévères
Pas de fatigue	❑	❑	❑	❑	❑	❑	❑	❑	❑	❑	❑	Fatigue sévère
Pas de difficultés respiratoires	❑	❑	❑	❑	❑	❑	❑	❑	❑	❑	❑	Difficultés respiratoires sévères
Pas d'état dépressif	❑	❑	❑	❑	❑	❑	❑	❑	❑	❑	❑	Etat dépressif sévère
Pas d'angoisse	❑	❑	❑	❑	❑	❑	❑	❑	❑	❑	❑	Angoisse sévère
Pas de confusion	❑	❑	❑	❑	❑	❑	❑	❑	❑	❑	❑	Confusion sévère
Pas de problème d'appétit	❑	❑	❑	❑	❑	❑	❑	❑	❑	❑	❑	Manque total d'appétit
Sensation de bien-être	❑	❑	❑	❑	❑	❑	❑	❑	❑	❑	❑	Mal-être profond
Pas de difficulté à se déplacer	❑	❑	❑	❑	❑	❑	❑	❑	❑	❑	❑	Confiné au lit

23 **Concernant cette personne, avez-vous pris la décision <u>d'intensifier le traitement de la douleur</u> et/ou des symptômes au moyen d'un ou plusieurs médicaments ?**

- ❑ Oui
- ❑ Non

23.1 **Quel(s) médicament(s) a(ont) été utilisé(s) ?**
(Plusieurs réponses possibles)

- ❑ Morphine ou autre opiacé
- ❑ Benzodiazépine
- ❑ Autres médicaments

23.2 **Aviez-vous pris en considération le fait que cette décision pouvait avancer la survenue de la mort du patient ?**

- ❑ Oui
- ❑ Non

23.3 **Aviez-vous l'intention délibérée d'avancer la survenue de la mort ?**

- ❑ Oui
- ❑ Non

23.4 **Pensez-vous que cette décision a avancé la survenue de la mort ?**

- ❑ Oui
- ❑ Non
- ❑ Je ne sais pas

22 **Concernant cette personne, avez-vous pris la décision d'arrêter un traitement susceptible de prolonger la vie ?**

- ❑ Oui
- ❑ Non

22.1 **Quel(s) traitement(s) a(ont) été arrêté(s) ?**
(Plusieurs réponses possibles)

- ❑ Hydratation artificielle
- ❑ Nutrition artificielle
- ❑ Ventilation artificielle
- ❑ Catécholamines
- ❑ Transfusion de produits sanguins
- ❑ Épuration extra-rénale
- ❑ Chimiothérapie, autre oncothérapie
- ❑ Chirurgie
- ❑ Autre traitement

22.2 **Aviez-vous pris en considération le fait que cette décision pouvait avancer la survenue de la mort ?**

- ❑ Oui
- ❑ Non

22.3 **Aviez-vous l'intention délibérée d'avancer la survenue de la mort ?**

- ❑ Oui
- ❑ Non

22.4 **Pensez-vous que cette décision a avancé la survenue de la mort ?**

- ❑ Oui
- ❑ Non
- ❑ Je ne sais pas

14 **Le patient a-t-il reçu des traitements à visées palliatives** *(soulagement des symptômes, traitement de la douleur)* **?**

- ❑ Oui
- ❑ Non
- ❑ Je ne sais pas

14.1 **Ces soins ont-ils été prodigués…**
(Plusieurs réponses possibles)

- ❑ à domicile par le médecin traitant et les soignants habituels
- ❑ à domicile avec intervention d'un réseau de soins palliatifs
- ❑ à domicile avec intervention d'un réseau de HAD (hospitalisation à domicile)
- ❑ à l'hôpital dans une unité de soins palliatifs
- ❑ à l'hôpital dans un service avec lit identifié "soins palliatifs"
- ❑ à l'hôpital dans un service sans lit identifié "soins palliatifs"
- ❑ à l'hôpital avec intervention d'une équipe mobile de soins palliatifs
- ❑ dans un établissement médico-social par le médecin traitant et les soignants habituels
- ❑ dans un établissement médico-social avec intervention d'un réseau de soins palliatifs
- ❑ dans un établissement médico-social avec intervention d'une équipe mobile de soins palliatifs
- ❑ autre

15 **Au moment du décès, le patient avait-il un traitement altérant sa vigilance ou sa conscience ?**

- ❑ Oui
- ❑ Non
- ❑ Je ne sais pas

15.1 **Quel(s) médicament(s) a (ont) été utilisé(s) ?**
(Plusieurs réponses possibles)

- ❑ Morphine ou autre opiacé
- ❑ Midazolam
- ❑ Autre benzodiazépine
- ❑ Propofol
- ❑ Autre médicament

15.2 **À quel moment, avant le décès, a-t-on débuté le traitement altérant sa vigilance ou sa conscience ?**

- ❑ Quelques heures
- ❑ Quelques jours
- ❑ Quelques semaines

15.3 **Ce traitement altérant sa vigilance ou sa conscience a-t-il été administré…**

- ❑ de manière continue
- ❑ de manière discontinue

15.4 **Ce traitement altérant sa vigilance ou sa conscience a-t-il été administré…**
(Plusieurs réponses possibles)

- ❑ en sachant qu'il n'avancerait pas la survenue de la mort
- ❑ en sachant qu'il pourrait éventuellement avancer la survenue de la mort
- ❑ sans se préoccuper qu'il pourrait éventuellement avancer la survenue de la mort
- ❑ dans le but explicite d'avancer la survenue de la mort
- ❑ dans le but de faire disparaître la perception d'une situation vécue comme insupportable par le patient
- ❑ dans un autre but

16 Le patient a-t-il reçu une hydratation artificielle ?

- ❑ Oui, de manière continue jusqu'au décès
- ❑ Oui, mais elle a été interrompue quelques heures avant le décès
- ❑ Oui, mais elle a été interrompue quelques jours avant le décès
- ❑ Oui, mais elle a été interrompue quelques semaines avant le décès
- ❑ Non
- ❑ Je ne sais pas

17 Le patient a-t-il reçu une nutrition artificielle ?

- ❑ Oui, de manière continue jusqu'au décès
- ❑ Oui, mais elle a été interrompue quelques heures avant le décès
- ❑ Oui, mais elle a été interrompue quelques jours avant le décès
- ❑ Oui, mais elle a été interrompue quelques semaines avant le décès
- ❑ Non
- ❑ Je ne sais pas

18 Qui était présent lorsque le patient est décédé ?

(Plusieurs réponses possibles)

- ❑ Vous ou un autre médecin
- ❑ Un(e) infirmier(e) ou un(e) aide-soignant(e)
- ❑ Un(e) garde malade
- ❑ Le conjoint
- ❑ Un (des) membre(s) de la famille
- ❑ Un (des) ami(s)
- ❑ Autre
- ❑ Personne n'était présent
- ❑ Je ne sais pas

19 Un soutien par un psychologue a-t-il été mis en place au cours du dernier mois avant le décès ?

(Plusieurs réponses possibles)

- ❑ Oui, pour le patient
- ❑ Oui, pour la famille
- ❑ Oui, pour les soignants
- ❑ Non
- ❑ Je ne sais pas

Nous allons maintenant aborder des questions qui requièrent une attention particulière en raison des réalités complexes qu'elles évoquent. Elles concernent plus précisément les différentes décisions en fin de vie prises par vous ou un autre médecin

20 Concernant cette personne, avez-vous d'abord décidé de <u>tout mettre en œuvre</u> pour empêcher la survenue de la mort ?

- ❑ Oui →
- ❑ Non

20.1 Quel(s) traitement(s) a(ont) été mis en œuvre ?

(Plusieurs réponses possibles)

- ❑ Hydratation artificielle
- ❑ Nutrition artificielle
- ❑ Ventilation artificielle
- ❑ Catécholamines
- ❑ Transfusion de produits sanguins
- ❑ Épuration extra-rénale
- ❑ Chimiothérapie, autre oncothérapie
- ❑ Chirurgie
- ❑ Autre traitement

21 Concernant cette personne, avez-vous pris la décision de <u>vous abstenir de mettre en oeuvre</u> un traitement susceptible de prolonger la vie ?

- ❑ Oui →
- ❑ Non

21.1 Quel(s) traitement(s) n'a(ont) pas été mis en œuvre ?

(Plusieurs réponses possibles)

- ❑ Hydratation artificielle
- ❑ Nutrition artificielle
- ❑ Ventilation artificielle
- ❑ Catécholamines
- ❑ Transfusion de produits sanguins
- ❑ Épuration extra-rénale
- ❑ Chimiothérapie, autre oncothérapie
- ❑ Chirurgie
- ❑ Autre traitement

21.2 Aviez-vous pris en considération le fait que cette décision pouvait avancer la survenue de la mort ?

- ❑ Oui
- ❑ Non

21.3 Aviez-vous l'intention délibérée d'avancer la survenue de la mort ?

- ❑ Oui
- ❑ Non

21.4 Pensez-vous que cette décision a avancé la survenue de la mort ?

- ❑ Oui
- ❑ Non
- ❑ Je ne sais pas
