# Supplementary material for: End-of-life medical decisions in France: a death certificate follow-up survey 5 years after the 2005 act of parliament on patients’ rights and end of life
Source: BMC Palliat Care. 2012 Dec 3;11:25. doi: 10.1186/1472-684X-11-25 (PMC3543844; doi:10.1186/1472-684X-11-25)
Supplement: Additional file 2 — Questionnaire of the French survey on End-of-life. [file 1472-684X-11-25-S2.pdf]

Key questions regarding medical end-of-life decisions in the French survey on end of life (La Fin de vie en France 2010)

1. Did you first of all do everything possible to prevent the death?

2. Did you take the decision to withhold a treatment that could have prolonged life?
- *a. Did you take into account the fact that this decision may hasten the death?*
- *b. Did you have the deliberate intention to hasten the death?*
- *c. Do you think this decision has hastened the death?*

3. Did you take the decision to withdraw a treatment that could have prolonged life?
- *a. Did you take into account the fact that this decision may hasten the death?*
- *b. Did you have the deliberate intention to hasten the death?*
- *c. Do you think this decision has hastened the death?*

4. Did you take the decision to intensify the treatment of pain and/or symptoms with one or more medication?
- *a. Did you take into account the fact that this decision may hasten the death?*
- *b. Did you have the deliberate intention to hasten the death?*
- *c. Do you think this decision has hastened the death?*

5. Did you take the decision to use one or more medications to deliberately end the person's life?

[1] Situation in which a third party intentionally ends a person' life, at his/her request, in order to end a situation that the person considers to be intolerable (ONFV, 2012)
